# Sarcosine Suppresses Epileptogenesis in Rats With Effects on Hippocampal DNA Methylation

**DOI:** 10.3389/fnmol.2020.00097

**Published:** 2020-06-05

**Authors:** Hai-Ying Shen, Landen Weltha, John M. Cook, Raey Gesese, Wakaba Omi, Sadie B. Baer, Rizelle Mae Rose, Jesica Reemmer, Detlev Boison

**Affiliations:** RS Dow Neurobiology Laboratories, Department of Translational Neuroscience, Legacy Research Institute, Portland, OR, United States

**Keywords:** epileptogenesis, sarcosine, DNA methylation, dentate gyrus, GlyT1 inhibition, TET1, DNMT

## Abstract

Epileptogenesis is a common consequence of brain insults, however, the prevention or delay of the epileptogenic process remains an important unmet medical challenge. Overexpression of glycine transporter 1 (GlyT1) is proposed as a pathological hallmark in the hippocampus of patients with temporal lobe epilepsy (TLE), and we previously demonstrated in rodent epilepsy models that augmentation of glycine suppressed chronic seizures and altered acute seizure thresholds. In the present study we evaluated the effect of the GlyT1 inhibitor, sarcosine (aka N-methylglycine), on epileptogenesis and also investigated possible mechanisms. We developed a modified rapid kindling model of epileptogenesis in rats combined with seizure score monitoring to evaluate the antiepileptogenic effect of sarcosine. We used immunohistochemistry and Western blot analysis for the evaluation of GlyT1 expression and epigenetic changes of 5-methylcytosine (5mC) and 5-hydroxymethylcytosine (5hmC) in the epileptogenic hippocampi of rats, and further evaluated expression changes in enzymes involved in the regulation of DNA methylation, ten-eleven translocation methylcytosine dioxygenase 1 (TET1), DNA-methyltransferase 1 (DNMT1), and DNMT3a. Our results demonstrated: (i) experimental evidence that sarcosine (3 g/kg, i.p. daily) suppressed kindling epileptogenesis in rats; (ii) the sarcosine-induced antiepileptogenic effect was accompanied by a suppressed hippocampal GlyT1 expression as well as a reduction of hippocampal 5mC levels and a corresponding increase in 5hmC; and (iii) sarcosine treatment caused differential expression changes of TET1 and DNMTs. Together, these findings suggest that sarcosine has unprecedented disease-modifying properties in a kindling model of epileptogenesis in rats, which was associated with altered hippocampal DNA methylation. Thus, manipulation of the glycine system is a potential therapeutic approach to attenuate the development of epilepsy.

## Introduction

Prevention of epileptogenesis remains an unmet medical challenge because underlying mechanisms involved in this process remain unclear (Pitkanen and Lukasiuk, 2011; Terrone et al., 2016). No effective treatment is currently available to prevent or delay the epileptogenic process while various therapeutic strategies were explored recently (Rowles and Olsen, 2012; Kaminski et al., 2014; Tchekalarova et al., 2014; Guo et al., 2015; Rensing et al., 2015; Siniscalchi and Mintzer, 2015; Castro et al., 2017; Long et al., 2017; Namvar et al., 2017; Talos et al., 2018; Upadhya et al., 2018, 2019). Epigenetic modification may play an important role in epileptogenesis (Hsieh and Gage, 2005; Kobow and Blumcke, 2012) and in particular global hypermethylation was identified in the epileptic hippocampus (Zhu et al., 2012). Such epigenetic changes can modify pathophysiological events in epileptogenesis and is also linked to the resistance to antiepileptic drugs (AEDs) (Kobow et al., 2013). These works support a role for methylation in the development and maintenance of epilepsy.

The dentate gyrus (DG) is a crucial brain region involved in the initiation or propagation of seizures (Dudek and Sutula, 2007; Sloviter et al., 2012) in temporal lobe epilepsy (TLE) due to its gating role from its densely packed, hyperpolarized granule cells (Toader et al., 2013). Our previous work demonstrated robust overexpression of glycine transporter 1 (GlyT1) in the hippocampus of human TLE samples and the DG region of epileptic rodents (Shen et al., 2015). Exogenous glycine administration can suppress neuronal excitation in the DG (Chattipakorn and McMahon, 2003) and reduce action potential firing in hippocampal neurons (Song et al., 2006). Augmentation of glycine has antiictogenic effects to suppress chronic seizures and alter the acute seizure threshold in rodents (Shen et al., 2015). Sarcosine (aka N-methylglycine), is a glycine transporter 1 (GlyT1) inhibitor that can increase extracellular glycine (Lopez-Corcuera et al., 1998; Herdon et al., 2001) via blockade of GlyT1-mediated glycine uptake or through a hetero-exchange of sarcosine for glycine (Herdon et al., 2001). Sarcosine agonizes the glycine receptor (GlyR) via binding to its strychnine-sensitive site (Salceda and Aguirre-Ramirez, 2005; Zhang et al., 2009). In addition, sarcosine acts as a co-agonist of N-methyl-D-aspartate receptor (NMDAR) (Ryley Parrish et al., 2013). Dysfunctional NMDAR signaling may contribute to the development of seizures and epilepsy (Barker-Haliski and White, 2015; Parrish et al., 2018). Finally, as an essential intermediate in one-carbon metabolism, sarcosine is involved in methyl balance and transmethylation fluxes (Ueland et al., 2007) and contributes to the dynamic homeostasis of transmethylation (Mudd et al., 2007; Moore et al., 2013). However, whether sarcosine’s actions through transmethylation have any relationship to seizure outcomes and epileptogenesis has not been previously evaluated.

Because status epilepticus can result in a hypermethylation status in the epileptogenic hippocampus (Miller-Delaney et al., 2013, 2015) and sarcosine is tightly linked to transmethylation pathways in the brain beyond its GlyT1 inhibition, thus sarcosine application may provide a novel strategy to ameliorate several aspects of epileptogenesis. We therefore hypothesized that manipulation of the glycine pathway via sarcosine may alter the progress of epileptogenesis via the transmethylation process and glycine-mediated inhibitory actions in the hippocampus. In the present study, we developed a rapid model of epileptogenesis for the purpose of establishment of a fast, stable, and reliable model to efficiently test the antiepileptic effects of pharmacological interventions. We then evaluated the potential antiepileptogenic role of sarcosine and explored epigenetic changes in the DG neurons for possible contributing mechanisms.

## Materials and Methods

### Animals

All animal procedures were conducted in a facility accredited by the Association for the Assessment and Accreditation of Laboratory Animal Care in accordance with protocols approved by the Institutional Animal Care and Use Committee of the Legacy Research Institute and the principles outlined by the National Institutes of Health (NIH). Male Sprague Dawley rats (280–300 g) were purchased from Jackson Labs (Sacramento, CA, United States). All rats were acclimatized for 1 week before being used in the experiments and the animals were housed in a temperature- and humidity-controlled room with a 12 h light/dark cycle (lights on at 7:00 a.m.) throughout the experimental period.

### Rat Kindling Model of Epileptogenesis

**FIGURE 1:**
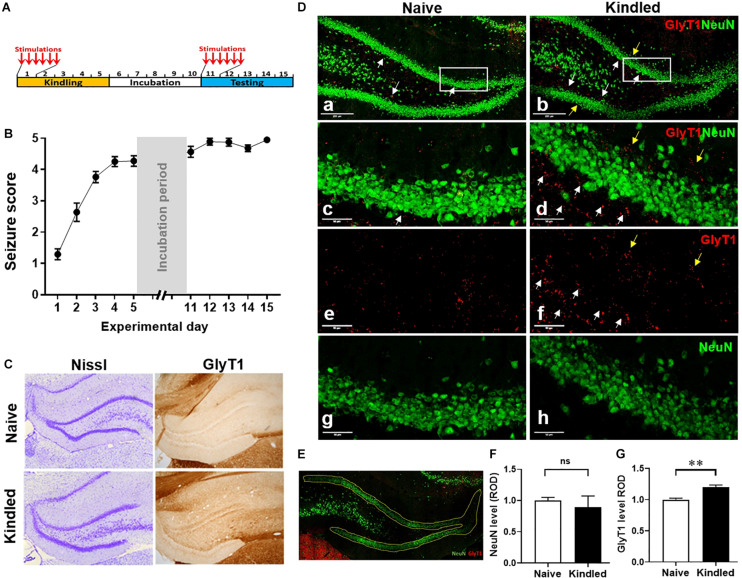
Hippocampal kindling induces overexpression of GlyT1. **(A)** Rat kindling paradigm that consists of a 5-day kindling period (days 1 –5), 5-day stimulation free incubation period (days 6–10), and a 5-day (re-kindling) testing period (days 11–15). **(B)** Averaged Racine stages of rats during their kindling and testing days. Data are mean ± SEM from six stimulations of each day. **(C)** Representative images of Nissl and GlyT1 immunochemistry DAB staining of fully kindled rats vs. non-kindled naïve controls. **(D)** Representative images of IF staining for GlyT1 and NeuN in the DG of fully kindled rats vs. non-kindled controls. **(E)** A representative selection of the DG area for the quantitative analysis of target positive staining. **(F)** Quantitative analysis of the DG expression levels of NeuN, or **(G)** GlyT1 in kindled rats (Kindled) vs. non-kindled controls (Naïve). White arrows pointing to dentate inner molecular layer, yellow arrows pointing to the dentate outer molecular layer. Data are mean ± SEM. **p < 0.01 vs. non-kindled controls, ns, no significance. Scale bar = 250 μm (a,b) or 50 μm (c–h).

The rat rapid electrical hippocampal kindling model of epileptogenesis was developed with modification from published kindling protocols (McNamara, 1986; Morimoto et al., 2004; De Smedt et al., 2007; Morales et al., 2014) to test drug effects on the process of epileptogenesis (paradigm see Figure 1A). Adult male Sprague Dawley rats were implanted with bipolar, coated stainless steel electrodes (0.20 mm in diameter; Plastics One, Roanoke, VA, United States) into the left hippocampus using the following stereotactic coordinates (*AP* = −5 mm, ML = +5 mm, DV = −7.5 mm, to bregma) and fixed with a headset of dental acrylate. All surgical procedures were performed under anesthesia induced with 3% isoflurane, 67% N_2_, 30% O_2_ and maintained with 1.5% isoflurane, 68.5% N_2_, 30% O_2_, while rats were placed in a Kopf stereotactic frame (David Kopf Instruments, CA, United States).

After recovery for 10 days, the rats were kindled based on a rapid kindling paradigm that consisted of three periods: 5-day kindling period, 5-day stimulus-free incubation period, and 5-day testing period. Briefly, using a Grass S-88 stimulator (Grass Telefa, United States), rats received six stimulations daily (1-ms square wave biphasic pulses of 200 μA, 50 Hz frequency, and 10 s duration at an interval of 30 min between stimulations) for 5 consecutive days. Behavioral seizures were scored according to the scale of Racine (1978), and electrical brain activity was amplified (Grass Technologies) and digitized (PowerLab; AD Instruments) for periods spanning one min prior- and five min post-application of each stimulating pulse. Following a 5-day stimulus-free incubation period, rats received six stimulations (as described above) daily for another 5 consecutive days (testing period) to evaluate the progression of epileptogenesis evidenced by their Racine scores post-stimulation.

For pharmacological experiments, rats were kindled as described above while being treated with sarcosine (SRC, 3 g/kg; #131776, Aldrich, CA, United States), valproic acid (VPA, 200 mg/kg; P4543, Sigma, CA, United States), 5-Aza-2′-deoxycytidine (5-AZA, 1.2 mg/kg; A3656, Sigma, CA, United States), or 0.9% saline (as vehicle control). Drugs were administered intraperitoneally (i.p.) 30 min prior to the first kindling session of each day for 5 consecutive days. After each stimulation, animals were scored for Racine stages to demonstrate and compare the seizure response in the presence of each experimental drug. Following a drug- and stimulus-free 5-day incubation period, rats received kindling stimuli and again were scored for Racine stages to evaluate the drugs’ antiepileptogenic potential. Rats were sacrificed at defined experimental time points (6, 11, or 16 days after initiation of kindling), with non-kindled rats as controls. Brains were either perfused for immunohistochemistry evaluation or fresh dissected for Western blot assessment.

### Immunohistochemistry Evaluation

Under anesthesia (with 68.5% N_2_, 30% O_2_, and 1.5% isoflurane), rats were transcardially perfused with ice-cold PBS followed by 4% formaldehyde. Brains were subsequently removed and post-fixed in 4% formaldehyde and then washed in PBS, infiltrated with a 30% sucrose PBS solution for cryoprotection. 30 μm coronal sections were sectioned using a cryostat (VT 1000S, Leica, Bannockburn, IL, United States). Sections were incubated for 1 h in 2% donkey blocking buffer (DBB, made of PBS containing 2% NDS, 1% BSA, and 0.02% Triton X-100), then incubated for 24 h at 4°C in blocking buffer containing primary antibodies, GlyT1, 5-methylcytosine (5mC), 5-hydroxy methylcytosine (5hmC), neuronal nuclei (NeuN), and glial fibrillary acidic protein (GFAP), as listed in Table 1. Then, for 3,3’-diaminobenzidine tetrahydrochloride (DAB) staining of GlyT1 (Shen et al., 2015), biotinylated goat anti-rabbit IgG (1:200, GE Healthcare, Diegem, Belgium) was used as the secondary antibody and the ABC mix (Vectastain ABC kit, Peroxidase Standard pk-4000, Vector Laboratories, Burlingame, CA, United States) coupled with 50 mg DAB peroxidase (HRP) substrate (SK-4105, Vector Labs) was used for detection. For immunofluorescence staining (Chouliaras et al., 2013), after several washes in PBS, sections were incubated with a blocking buffer containing corresponding fluorochrome-conjugated secondary antibodies in Table 1 for detection. The sections were then washed and mounted using the mounting medium with DAPI for fluorescence microscopy observation on a Leica TCS SPE confocal laser-scanning microscope (LAS X 3.1.2.16221). All sections were processed in parallel using identical solutions and identical incubation times, while slices without either primary or secondary antibody were used for controls.

**TABLE 1:** Antibodies used for immunohistochemistry and Western blot evaluation.

Antibody	Catalog #	Manufacturers	Dilution
GlyT1	n/a	In house (Shen HY, 2015)*	1:250 (IHC)
5mC	A-1014	Epigentek, Farmingdale, NY	1:2000 (IHC)
5hmC	39769	Active Motif, Carlsbad, CA	1:3000 (IHC)
NeuN	MAB-377	Millipore Sigma, Burlington, MA	1:500 (IHC)
GFAP	NB100-53809	Novus Biologicals, Centennial, CO	1:1000 (IHC)
TET1	PA5-72805	Thermo Fisher Scientific, Waltham, MA	1:1000 (WB)
DNMT1	D63A6	Cell signaling, Beverly, MA	1:1000 (WB)
DNMT3a	3598S	Cell signaling, Beverly, MA	1:1000 (WB)
α-tubulin	sc-8035	Santa Cruz Biotechnology, CA, USA	1:5000 (WB)
Alexa Fluor 488 donkey anti-mouse IgG	A21202	Life Technologies, Carlsbad, CA	1:500 (IHC)
Alexa Fluor 555 donkey anti-rabbit IgG	A31572	Life Technologies, Carlsbad, CA	1:350 (IHC)
Alexa Fluor 633 donkey anti-goat IgG	A21082	Life Technologies, Carlsbad, CA	1:1000 (IHC)
Alexa Fluor 488 goat anti-mouse IgG	A1101	Life Technologies, Carlsbad, CA	1:400 (IHC)
Alexa Fluor 555 goat anti-rabbit IgG	A21428	Life Technologies, Carlsbad, CA	1:400 (IHC)

GlyT1, glycine transporter 1; 5mC, 5-methylcytosine; 5hmC, 5-hydroxy methylcytosine; NeuN, neuronal nuclei; GFAP, glial fibrillary acidic protein; TET1, ten-eleven translocation methylcytosine dioxygenase 1; DNMT1 and DNMT3a, DNA methyltransferase 1 and 3a; GAPDH, Glyceraldehyde 3-phosphate dehydrogenase; IHC, immunohistochemistry; WB, Western blot. *Reference: Shen et al. (2015).

### Image Quantification of Densitometry

High-resolution digital images were acquired under identical conditions, and all image processing was applied identically across experimental groups. The images of immunofluorescence staining were acquired using the LasX software system (Leica, Buffalo Grove, IL, United States). Fluorescence intensity analysis was performed using Leica Application Suite Analysis software (Leica, Buffalo Grove, IL, United States). The dentate gyrus was selected using binary processing of the LasX software system (Leica, Buffalo Grove, IL, United States) as shown in Figure 1E, and immunoreactive material was measured in the same designated area of the DG for each sample and expressed as relative optical density (ROD) by area. Three levels were measured for each rat, and data analysis is expressed as the mean ± SEM of ROD. The average levels in treatment groups were normalized according to that in the control group (as baseline).

### Western Blot Assay

Hippocampi were freshly dissected after animals were sacrificed at designed experimental time points, brain tissue was processed for the extraction of aqueous membrane proteins and a 30 μg sample of protein from each specimen was loaded and electrophoresed on a 10% Bis-Tris gel. After the transfer of the proteins onto nitrocellulose membranes, the blots were blocked for 1 h in TBSTM (10 mM Tris/HCl, pH 8, 0.15 M NaCl, 0.05 % Tween 20, containing 5% nonfat milk) at room temperature, followed by incubation with primary antibodies (Table 1): anti-ten-eleven translocation methylcytosine dioxygenase 1 (TET1), anti-DNA methyltransferase1 (DNMT1), or anti-DNMT3a, overnight at 4°C in TBSTM, 4 × 10 min washes with TBST and incubation with corresponding secondary antibodies (#7074, 1:8,000, Cell Signaling, Boston, MA, United States) at RT for 1 h. After 4 × 10 min washing with TBST, immunoreactivity was detected by chemiluminescence (#34087, Pierce, IL, United States); the immunoblots were quantified using a ChemiDoc Touch Imaging System (#17001401, Bio-Rad, Hercules, CA, United States). To normalize protein loading, a mouse monoclonal anti-α-tubulin antibody (sc-8035, Santa Cruz Biotechnology, CA, United States) was used to re-probe the same blot and the OD ratio of ADK to α-tubulin was calculated.

### Statistics

Quantitative data were analyzed using GraphPad Prism 8 software. All data are presented as mean ± SEM, and data were analyzed using one-way, two-way ANOVA, or *t*-tests assuming the non-Gaussian distribution of the data as appropriate. A *p* < 0.05 was considered significant.

## Results

### Hippocampal Kindling Induced Epileptogenesis and GlyT1 Overexpression in the Dentate Gyrus

We developed a rapid kindling model of epileptogenesis in rats (Figure 1A) to evaluate phenotypical and molecular changes in epileptogenesis. More than 90% of rats (11 out of 12) were fully kindled after 5-days of kindling, as defined by scoring a Racine seizure stage of 4–5 after the first test stimulus on each day of testing (Figure 1B). The histological evaluation of Nissl staining at experimental day 16 showed a similar morphology of neuronal cells in DG between fully kindled rats and non-kindled controls. In contrast, GlyT1-DAB staining demonstrated an overt increase of GlyT1-positive staining in the epileptogenic hippocampus (Figure 1C). Of note, the overexpressed GlyT1 was predominantly located at the dentate inner molecular layer, whereas a lesser increase was seen in the dentate outer molecular layer (Figure 1D). Quantitative analysis from DG-focused IF staining (Figure 1E) demonstrated that NeuN-positive staining in the DG was not significantly different between fully kindled rats and the non-kindled naive controls (*p* = 0.6476, unpaired *t*-test, *n* = 3–4 per group) (Figure 1F); this is in line with Nissl observations and strongly indicates that neuronal cell death is not a contributing pathophysiological feature of our rapid kindling model of epileptogenesis. Importantly, quantitative analysis from IF staining of GlyT1 demonstrated a significant (20.2%) increase of GlyT1 in the DG of fully kindled rats vs. non-kindled controls (*p* = 0.0066, unpaired *t*-test, *n* = 3–4 per group) (Figure 1G). These results, which are in line with our previous findings, demonstrate pathological increases in GlyT1 in the rapid rat hippocampal kindling model of epileptogenesis.

### Dysregulated DNA Methylation in the Epileptogenic DG of Kindled Rats

**FIGURE 2:**
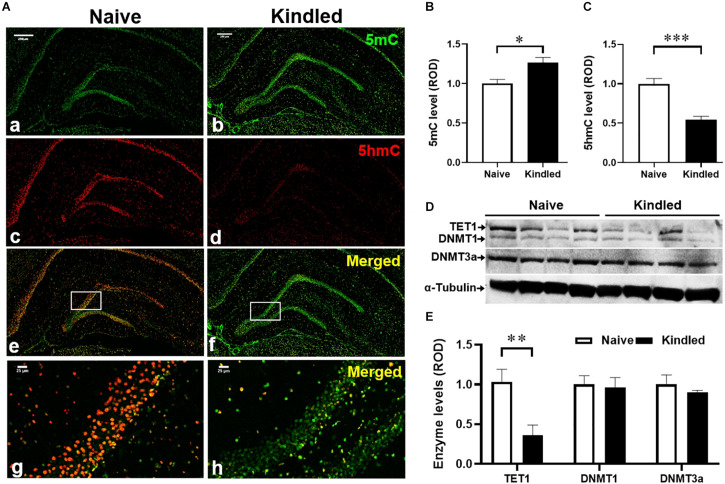
Changes of DNA methylation in kindling epileptogenesis. **(A)** Immunofluorescence staining for 5mC and 5hmC n the hippocampus of fully kindled rats vs. non-kindled naïve controls. Scale bar = 250 μm (a–f) and 25 μm (g,h). Quantitative analysis of the DG levels of **(B)** 5mC and **(C)** 5hmC in non-kindled naïve controls and fully kindled rats. **(D)** Representative Western blot imaging of TET1, DNMT1, and DNMT3a. **(E)** Quantification of hippocampal levels of TET1, DNMT1, and DNMT3a in fully kindled rats vs. non-kindled naïve controls. Data are mean ± SEM. *p < 0.05, **p < 0.01, ****p < 0.0001 vs. non-kindled naïve controls.

To explore potential epigenetic mechanisms underlying epileptogenesis in kindled rats, we evaluated the methylation changes in the DG of kindled vs. non-kindled rats. The IF staining showed that the expression levels of 5mC, a biomarker of DNA methylation, were significantly increased in the granule cell layer of the DG in kindled rats on experimental day 16 compared to non-kindled controls (*p* = 0.017, unpaired *t*-test, *n* = 4–5 per group) (Figures 2A,B). In contrast, the expression levels of 5hmC, a demethylation biomarker, were significantly decreased in the granule cell layer of the DG in kindled rats vs. non-kindled controls (*p* < 0.0001, unpaired *t*-test, *n* = 4–5 per group) (Figures 2A,C). These findings suggest an altered methylation status of 5mC and 5hmC in the epileptogenic brain. To validate and further probe mechanisms contributing to those findings, we assessed related changes of the expression levels of DNA methyltransferases DNMT1 and DNMT3a, and the demethylase TET1. Our Western blot data demonstrate that kindling-induced epileptogenesis resulted in differential response on expression between methylase DNMT1, DNMT3a, and demethylase TET1 [*p* = 0.0325, *F*_(__1, 9)_ = 6.374, kindling effect; *p* = 0.0463, *F*_(2, 9)_ = 4.408, enzyme effect; two-way ANOVA]. Specifically, the hippocampal expression of TET1 was significantly lower in fully kindled rats vs. controls (*p* = 0.0081, unpaired *t*-test, *n* = 4 per group) (Figures 2D,E), whereas expression levels of DNMT1 and DNMT3a were not significantly different between kindled and non-kindled rats (*p* = 0.4138 and *p* = 0.2237, correspondingly, unpaired *t*-test, *n* = 4 per group). The above findings suggest that DNA methylation is dysregulated in kindled rats.

### Sarcosine Effectively Suppresses Kindling Epileptogenesis

**FIGURE 3:**
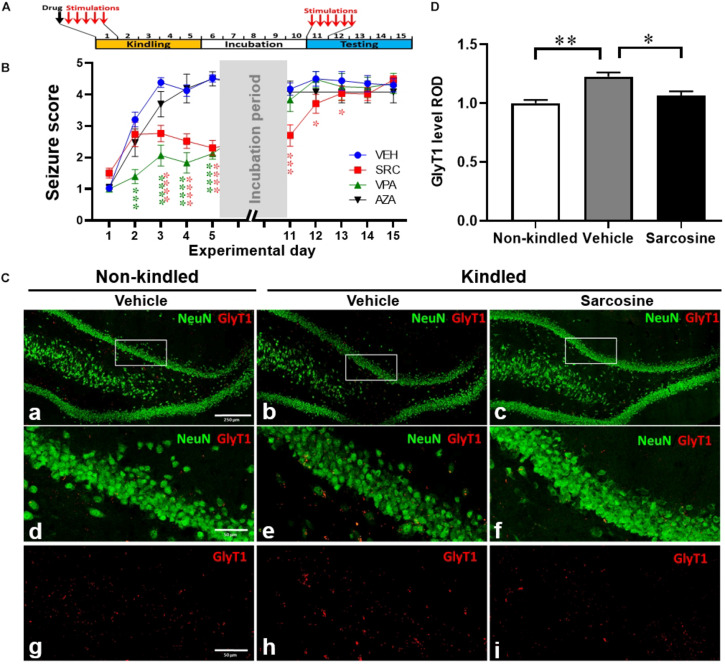
Sarcosine treatment during kindling attenuates seizures and kindling epileptogenesis. **(A)** Rat kindling and drug treatment paradigm. Drug treatments were given 30 min prior to each day’s first kindling stimulation. **(B)** Racine stages of treated rats during their kindling and testing days vs. vehicle-treated controls. Data are mean ± SEM from six stimulations of each day. *p < 0.05, ***p < 0.001, ****p < 0.0001 vs. vehicle-treated controls. **(C)** Representative IF staining of GlyT1 and NeuN in the DG of kindled rats with the treatment of sarcosine (3 g/kg, i.p.) vs. vehicle, sacrificed on day 11. **(D)** Quantification of DG levels of GlyT1 on day 11 in kindled rats that received 5 days of daily treatment of sarcosine or vehicle (*n* = 4–5 per group). *p < 0.05, **p < 0.01. Scale bar = 250 μm (a) and 50 μm (d,g).

Having identified a disease state of hypermethylation and dysregulated GlyT1 expression in epileptogenesis of rats, we used a separate cohort of rats to evaluate the potential antiepileptogenic actions of sarcosine and its effects on transmethylation. During the kindling period rats were given a single daily i.p. injection of the GlyT1 inhibitor sarcosine (SRC, 3 g/kg), a conventional AED, valproic acid (VPA, 200 mg/kg), the demethylating agent 5-azacytidine (5-AZA, 1.2 mg/kg), or 0.9% saline (as vehicle control) for 5 days, 30 min prior to each day’s first kindling stimulation (Figure 3A). Vehicle-treated rats (Figure 3B) (*n* = 15) rapidly reached an average Racine score of at least 4 at the end of the kindling period, and maintained high seizure responses during the testing period, confirming our established rapid-epileptogenic kindling model (as shown in Figure 1). SRC treatment significantly suppressed kindling-induced epileptogenesis [*p* < 0.0001, *F*_(__3, 46)_ = 17.31, treatment effect, *p* < 0.0001, *F*_(__9, 389)_ = 92.77, time effect; and *p* < 0.0001, *F*_(__27, 389)_ = 6.085, treatment × time effects; two-way ANOVA] with reduced seizure scores during kindling period, day 3, 4, and 5 (*p* < 0.0001, *t*-test, *n* = 30), and a lasting suppression of seizure responses during the testing period, day 11, 12, and 13 (*p* < 0.001, *p* = 0.0145, *p* = 0.0254, correspondingly, *t*-test, *n* = 17), which indicates SRC-induced anti-ictogenic and anti-epileptogenic effects. Treatment with the conventional AED drug VPA (200 mg/kg, *n* = 8) suppressed seizure responses during the kindling period but had no lasting effects during the testing period. Interestingly, treatment with the demethylating agent, 5-AZA, at a dose of 1.2 mg/kg showed neither an anti-ictogenic nor an anti-epileptogenic effect (*n* = 6) (Figure 3B). The above findings support a novel antiepileptogenic effect of sarcosine.

### Sarcosine Treatment Normalizes GlyT1 Expression in the Kindled Rats

To investigate whether sarcosine treatment during kindling affects GlyT1 expression, we evaluated the expression levels of GlyT1 in the DG of rats that received sarcosine treatment during the first 5 days of kindling (Figure 3C). The quantitative analysis from IF staining showed a significant treatment effect of sarcosine on the DG GlyT1 levels of kindled rats [*n* = 4–5 per group, *p* = 0.0021, *F*_(__2, 10)_ = 12.13, one-way ANOVA]. While GlyT1 levels in the DG of vehicle-treated kindled rats were significantly higher than in non-kindled controls (*p* = 0.0021, unpaired *t*-test), the sarcosine (3 g/kg, i.p.) treatment significantly suppressed the overexpression of DG GlyT1 (by 21.5%) compared to vehicle-treated kindled rats on day 11 (*p* = 0.0165, unpaired *t*-test) (Figure 3D). Thus, sarcosine treatment normalized GlyT1 expression levels in kindled rats, to levels similar to those found in non-kindled rats (*p* = 0.4601, unpaired *t*-test).

### Sarcosine Treatment Affects DNA Methylation in Kindled Rats

**FIGURE 4:**
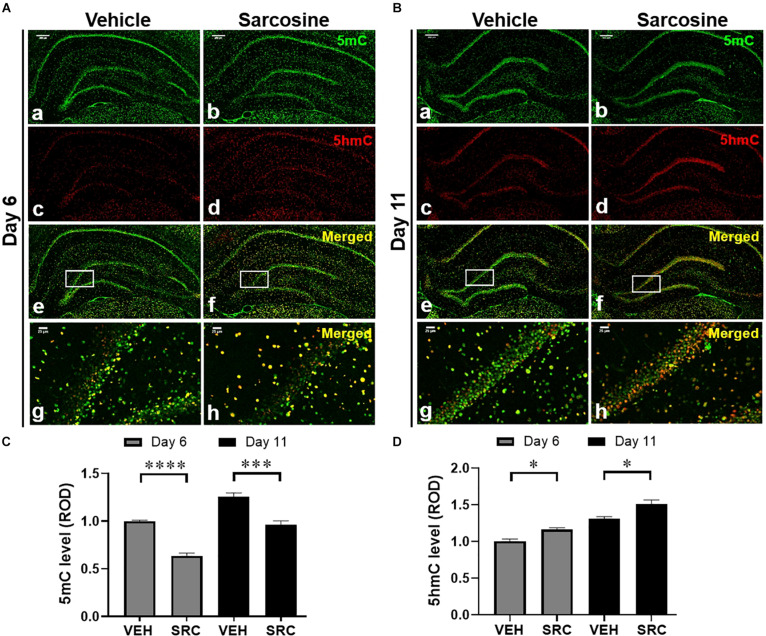
Sarcosine affects DNA methylation in kindling epileptogenesis. Immunofluorescence staining of 5mC and 5hmC in the hippocampus of kindled rats on day 6 **(A)** or day 11 **(B)**, which received sarcosine or vehicle treatment. Scale bar = 250 μm (a–f) and 50 μm (g,h). Quantitative analysis of the DG levels of 5mC **(C)** and 5hmC **(D)** in rats with sarcosine vs. vehicle treatment. *p < 0.05, ***p < 0.001, and ****p < 0.0001 vs. vehicle-treated controls.

To explore the effects of sarcosine administration during kindling on DNA methylation, we evaluated 5mC and 5hmC expression levels in the DG and assessed hippocampal expression levels of methylase and demethylase. Tissue samples were taken on day 6 (24 h after the last sarcosine treatment) (Figure 4A) and day 11 (after 5 days of drug washout and the stimulus-free period) (Figure 4B). The IF staining data showed that sarcosine treatment significantly reduced the 5mC levels in the DG vs. vehicle-treated controls (*n* = 4–5 per group) [*p* < 0.0001, *F*_(__1, 46__)_ = 52.91, treatment effect; *F*_(__1, 46)_ = 33.84, time effect, two-way ANOVA] on both day 6 and day 11 (*p* < 0.0001 and *p* = 0.0003, correspondingly, unpaired *t*-test) (Figure 4C). In contrast, the sarcosine treatment increased the DG 5hmC levels in rats treated with sarcosine vs. vehicle [*n* = 4–5 per group, *p* = 0.0005, *F*_(__1, 46__)_ = 14.07, treatment effect; *p* = 0.0005, *F*_(__1, 46)_ = 27.93, time effect, two-way ANOVA] on day 6 and day 11 (*p* = 0.0477 and *p* = 0.0127, correspondingly, unpaired *t*-test) (Figure 4D).

**FIGURE 5:**
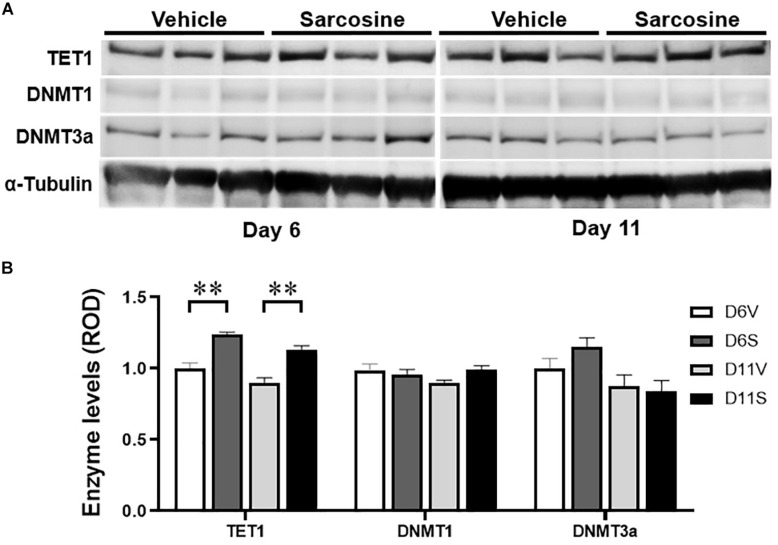
Sarcosine affects DNA methylase and demethylase. **(A)** Representative Western blot imaging of TET1, DNMT1, and DNMT3a of the hippocampus of rats exposed to sarcosine during kindling and dissected on experimental days 6 and 11. **(B)** Quantification of hippocampal levels of TET1, DNMT1, and DNMT3a in sarcosine-treated rats vs. vehicle-treated controls. Data are mean ± SEM. **p < 0.01 vs. controls.

In addition, our Western blot assay showed that sarcosine treatment increased hippocampal TET1 expression levels vs. vehicle-treated kindled rats [*p* = 0.0004, *F*_(__1, 10)_ = 27.54, treatment effect; *p* = 0.2687, *F*_(__2, 10)_ = 1.371, time effect; two-way ANOVA; *n* = 6 per group] on both day 6 and day 11 (*p* = 0.0080 and *p* = 0.0081, correspondingly, unpaired *t*-test) (Figures 5A,B, left panel), which correlated to increased DG 5hmC levels on day 6 and day 11. However, the hippocampal expression levels of DNMT3a were not significantly different between sarcosine- and vehicle-treated rats on either day 6 or day 11 [*p* = 0.6537, *F*_(__1, 10)_ = 0.2138, treatment effect; *p* = 0.6962, *F*_(__2, 10)_ = 0.6962, time effect; two-way ANOVA; *n* = 6 per group] (Figures 5A,B, middle panels). Similarly, the hippocampal levels of DNMT3a of the sarcosine-treated rats were not different from vehicle-treated rats on either day 6 or day 11 (Figures 5A,B, right panel) The above data showed that sarcosine altered DNA methylation in the DG of kindled rats, by decreasing 5mC and increasing 5hmC and TET1.

## Discussion

There exists an urgent and unmet clinical need to prevent or delay the development of epilepsy (Pitkanen and Lukasiuk, 2011; Di Maio, 2014; Younus and Reddy, 2017). Despite extensive studies exploring possible mechanisms of epileptogenesis (Rakhade and Jensen, 2009; Goldberg and Coulter, 2013; Engel and Pitkanen, 2019) and therapeutic strategies for reducing the impact of epileptogenesis (Rowles and Olsen, 2012; Kaminski et al., 2014; Tchekalarova et al., 2014; Guo et al., 2015; Rensing et al., 2015; Siniscalchi and Mintzer, 2015; Castro et al., 2017; Long et al., 2017; Namvar et al., 2017; Talos et al., 2018; Upadhya et al., 2018, 2019), no pharmacological interventions are currently available to reduce or delay the progression of epileptogenesis. In the present study, we developed a rapid hippocampal kindling model to produce stable epileptogenesis (Figure 1A) and demonstrated a resultant overexpression of GlyT1 and dysregulated DNA methylation in kindled rats. We further provided experimental evidence that sarcosine can delay kindling epileptogenesis, which was associated with altered DNA methylation changes and normalized GlyT1 expression levels. Sarcosine treatment during kindling changed hippocampal 5mC and 5hmC levels and modified the expression levels of the demethylase TET1. To better understand the role of sarcosine in epileptogenesis and its therapeutic potential, the following aspects of sarcosine warrant further discussion.

### Role of GlyT1 in the Dentate Gyrus During Kindling

The DG, as a primary source of seizure activity, has long been a focal point to study possible molecular, cellular, and network mechanisms responsible for epileptogenesis in TLE (Dudek and Sutula, 2007; Toader et al., 2013; Wei et al., 2015). While multiple mechanisms can contribute to epileptogenesis in acquired TLE, neuroprotection provided immediately after epileptogenic insults were proposed to be the most effective strategy against epileptogenesis (Sloviter et al., 2012). Recently we demonstrated pathological overexpression of GlyT1 in the epileptogenic DG of kindled rats. Thus, inhibition of GlyT1, by its inhibitor sarcosine was used as a rationale to both block GlyT1 directly and also act as a source of glycine, thereby increasing the availability of an inhibitory neuromodulator during epileptogenesis. Noteworthy, we observed that overexpressed GlyT1 in the epileptogenic DG was specifically located at the dentate inner molecular layer, which is innervated by *excitatory* hilar mossy cells, but not at the dentate outer molecular layer, which is innervated by *inhibitory* interneurons (Figure 1D). Overexpression of GlyT1 at the dentate inner molecular layer could lead to reduced availability of extracellular glycine and weakened activation of glycine receptor-mediated inhibition, which in turn, may exacerbate excitation of mossy cells, which may contribute to epileptogenesis. Indeed, sarcosine treatment during kindling reduced the expression of GlyT1 in the DG. However, full validation of the underlying mechanisms requires future work including electrophysiological studies for granule cell excitability in a subset of cell populations, and also hippocampal pyramidal cells.

### Sarcosine Affects DNA Methylation During Kindling

**FIGURE 6:**
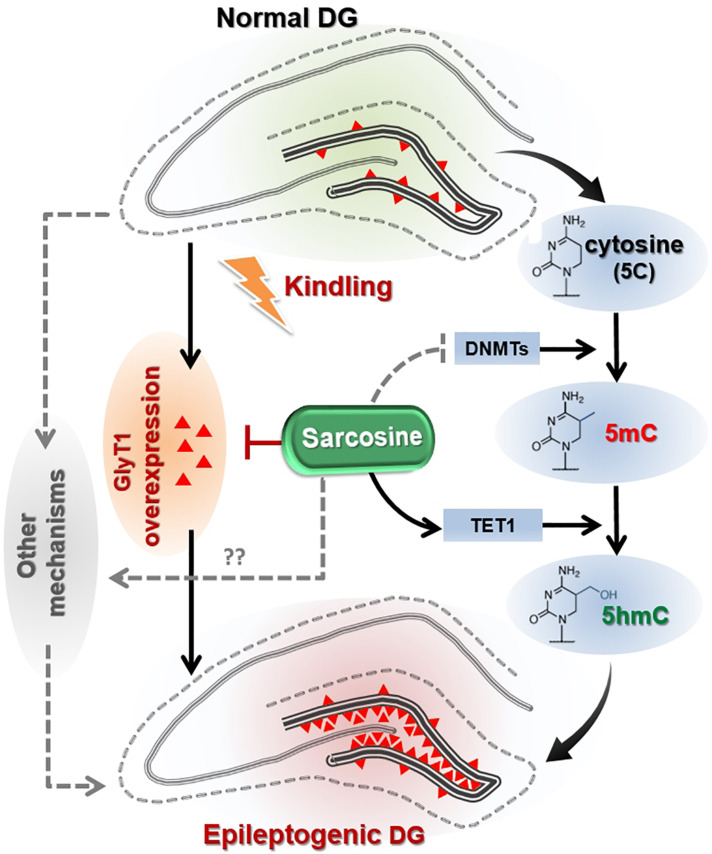
Hypothetical pathways linking sarcosine with antiepileptogenic activity. Kindling of the hippocampus in rats results in GlyT1 overexpression and DNA hypermethylation in the hippocampal dentate gyrus (DG), two putative mechanisms thought to promote epileptogenesis. Sarcosine can suppress the overexpression of GlyT1 in the epileptogenic DG and reduce the hypermethylation status via enhancement of TET1 expression and its demethylation process of converting 5mC into 5hmC.

Recent studies have indicated that DNA methylation is tightly linked to seizure development and epileptogenesis (Kobow et al., 2013; Wang et al., 2017). Overall, a general hippocampal DNA hypermethylation status appears to be associated with chronic epilepsy (Wang et al., 2016; Boison and Rho, 2019). At the same time, hypomethylation in specific genes was observed in (myoclonic) epilepsy, such as in genes encoding the Na-K-2Cl cotransporter isoform 1 (*NKCC1*) and the K-Cl cotransporter isoform 2 (*KCC2*), which affect accumulating and extruding chloride ion (Cl-) flows (Genc et al., 2019). The purpose of this study was not to perform a gene ontology analysis of those genes displaying differential methylation in the TLE model; rather, our study assessed global DNA methylation changes as possible epigenetic mechanisms linked to sarcosine, which might be mobilized for the suppression of epileptogenesis. First, we demonstrated an increase of 5mC in the epileptogenic dentate gyrus of kindled rats, whereas we failed to observe correlate changes in the hippocampal expression of the DNA methylases DNMT1 or DNMT3a. Importantly, a significant decrease in 5hmC was identified in the epileptic DG indicating a dysregulation in the demethylation process. Conversely, treatment with sarcosine during kindling increased the level of 5hmC in the DG and this effect was associated with a delay in the progression of epileptogenesis. Namely, sarcosine treatment resulted in a reduced global DNA methylation status likely linked to enhanced demethylation. The increase of TET1 expression in the DG of kindled rats treated with sarcosine may contribute to facilitated demethylation of DNA via the formation of increased 5hmC (Figure 6). It is worth noting that the lack of changes in hippocampal DNMT1 and/or DNMT3a levels could be due to Western blot evaluation, which lacks spatial resolution to reflect local changes in the DG. Taken together, our findings support our hypothesis that manipulation of the glycine pathway via sarcosine can modify epileptogenesis and epigenetic processes in the hippocampus. However, sarcosine-mediated DNA methylation changes seem to be unique, because our study showed that the conventional demethylating agent, 5-AZA, neither delayed epileptogenesis in kindled rats nor suppressed seizures during kindling. Differences between the effects of sarcosine and 5-AZA can best be explained by our findings suggesting an involvement of sarcosine in active DNA demethylation, whereas 5-AZA interferes with DNA methylation: e.g., the different targets between sarcosine (enhanced TET1) and 5-AZA (suppressed global methylation). It is therefore likely that any anti-epileptogenic effects of sarcosine which might be mediated via methylation status are likely nuanced and site-specific. In addition, in contrast to 5-AZA, sarcosine exerts multiple activities, such as GlyT1 inhibition and action on the glutamatergic signaling pathway as a co-agonist of NMDARs, which may synergistically or independently contribute to the antiepileptogenesis (Figure 6). Additional GlyT1 inhibitors, and/or epileptogenesis models are warranted to further the unique features of sarcosine. Indeed, in a pilot study, lower doses of sarcosine (1 and 2 g/kg) were unable to delay kindling epileptogenesis or suppress seizures during kindling (data not shown).

## Challenges and Remarks

As prevention of epileptogenesis is an unmet medical challenge, there have been many studies attempting to repurpose current AEDs to explore their antiepileptogenic potential (Pitkanen, 2010; Pitkanen et al., 2013; Loscher and Friedman, 2020). However, most attempts have had less than satisfactory outcomes to delay or slow down the progression of epilepsy (Lukawski et al., 2018). As shown in this study, a conventional AED, valproic acid, only acutely suppressed the kindling ictogenic seizures, but did not affect epileptogenesis. In contrast, sarcosine effectively suppressed ictogenic seizures and suppressed kindling epileptogenesis, indicating a unique feature of GlyT1 inhibition in the treatment of epilepsy. However, several limitations can be addressed in future studies: for instance, the behavioral aspects of spontaneous status epilepticus were not monitored in the present study; rather, the response seizure score to kindling was used as the major index to study epileptogenesis. Also, the electrophysiological evaluation of spontaneous activity within the granule cell layer should be included in future studies, which will extend our understanding of sarcosine and other GlyT1 inhibitors on epileptogenesis. Finally, the epigenetic studies need to be expanded to assess specific DNA methylation changes at individual target genes. In conclusion, our findings suggest that manipulation of the glycine system represents a novel and promising approach to preventing or minimizing epileptogenesis.

## Data Availability Statement

The datasets generated for this study are available on request to the corresponding author.

## Ethics Statement

The animal study was reviewed and approved by Legacy Research Institute.

## Author Contributions

H-YS and DB: conception and experimental design. LW, JC, RG, WO, SB, and HY-S: acquisition of data or material support. JC, RG, H-YS, JR, and RR: analysis and interpretation of data. H-YS: manuscript writing.

## Conflict of Interest

The authors declare that the research was conducted in the absence of any commercial or financial relationships that could be construed as a potential conflict of interest.
